# Exploiting Natural Products to Build Metalla-Assemblies: The Anticancer Activity of Embelin-Derived Rh(III) and Ir(III) Metalla-Rectangles

**DOI:** 10.3390/molecules19056031

**Published:** 2014-05-12

**Authors:** Gajendra Gupta, Jerald Mahesh Kumar, Amine Garci, Narayana Nagesh, Bruno Therrien

**Affiliations:** 1 Institute of Chemistry, University of Neuchatel, Avenue de Bellevaux 51, CH-2000 Neuchatel, Switzerland; E-Mails: gajendra.gupta@unine.ch (G.G.); amine.garci@unine.ch (A.G.); 2 CSIR-Centre for Cellular and Molecular Biology, Uppal Road, Habsiguda, Hyderabad-500 007, India; E-Mail: mahesh73@ccmb.res.in

**Keywords:** bioinorganic chemistry, metalla-assemblies, coordination-driven self-assembly, anticancer activity, half-sandwich complexes

## Abstract

Six new pentamethylcyclopentadienyl Rh(III) and Ir(III) metalla-rectangles ([**3**](CF_3_SO_3_)_4_–[**8**](CF_3_SO_3_)_4_) have been prepared by a self-assembly strategy using the embelin-derived metalla-clips (η^5^-C_5_Me_5_)_2_M_2_(μ_4_-C_6_HRO_4_-κ*O*)Cl_2_ (M = Rh, **1**; M = Ir, **2**; R = (CH_2_)_10_CH_3_) and the linear ditopic ligands, pyrazine, 4,4'-bipyridine and 1,2-bis(4-pyridyl)ethylene. These new metalla-rectangles have been obtained in high yield and isolated as their triflate salts. The complexes have been fully characterized by standard spectroscopic techniques and the antiproliferative activity of these tetranuclear complexes was evaluated *in vitro* on cancerous (DU-145, A-549, HeLa) and noncancerous (HEK-293) cell lines. The biological study has showed a better activity for the rhodium derivatives over the iridium analogs and for all complexes a very good selectivity for cancerous over noncancerous cells. The presence of lipophilic side chains coupled with the positive charge of the tetranuclear complexes suggested a cytotoxic activity involving the mitochondrial machinery, as demonstrated by multiple biological experiments.

## 1. Introduction

Plant extracts offer an infinite supply of biological agents. For centuries, humans have used plant extracts to cure diseases. However, identifying, purifying and isolating the biologically active compounds from plant extracts remain a difficult task, and too often, after this laborious work, the compounds appear to be of limited used for physico-chemical reasons, such as poor solubility, poor selectivity, or poor stability [1]. Therefore, coupling an organic drug with an organometallic entity can potentially alleviate the undesirable physico-chemical properties of a natural product and accordingly generate a much powerful drug [2]. This has been nicely demonstrated with ferroquine [3], a successful organometallic antimalarial drug combining chloroquine and ferrocene, which shows activity against chloroquine-resistant malaria strains.

**Figure 1:**
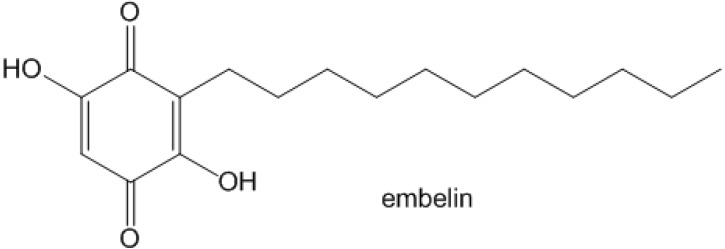
Molecular structure of embelin.

Among natural products, phenolic lipids are an interesting family of derivatives that includes catechol, resorcinol and hydroquinone [4]. Mainly extracted from plants, phenolic lipids are generally considered to be secondary metabolites (substances not essential for cell growth) [5]. Embelin is a phenolic lipid extracted from plants of the *Myrsinaceae* family [5]. Embelin shows an amphiphilic nature, having an alkyl chain and a dihydroxybenzoquinone unit (Figure 1). The alkyl chain increases cell permeability, and embelin itself has been found to inhibit X-linked inhibitors of apoptosis proteins (XIAP) [6]. XIA-proteins tend to interact with caspase-9, an important initiator of apoptosis, thus helping cancer cells to become immortal by blocking their programmed cell death [7]. Therefore, allowing cancer cells to regain their ability to proceed to apoptosis by inhibiting XIAP is an elegant strategy to fight cancers.

Recently, coordination-driven self-assemblies have made an entry in biology [8]. Metalla-cycles and metalla-cages are now employed to stabilize DNA motifs, to interact with proteins, or to transport guest molecules to cells [9,10,11]. The construction of metalla-assemblies involves various types of ligands, which depend on the choice of the metal centers and on the anticipated architectures. When dealing with three-legged piano-stool complexes, a strategy has emerged from the group of Süss-Fink [12], in which two oxalato-bridged dinuclear *p*-cymene ruthenium complexes were assembled with two 4,4'-bipyridine linear connectors to afford a tetracationic metalla-rectangle. This strategy is now widely used to generate two- and three-dimensional arene ruthenium assemblies [13], and it has been extended to half-sandwich rhodium and iridium complexes [14].

**Figure 2:**
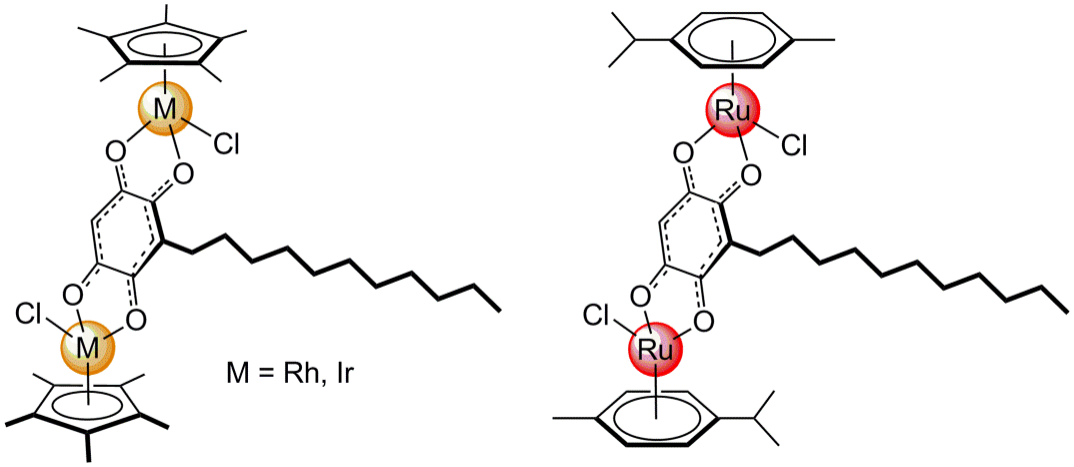
Molecular structure of embelin-derived dinuclear complexes.

Embelin possesses four oxygen atoms that can be used to synthesize (μ_4_-C_6_HRO_4_-κ*O*) (R = (CH_2_)_10_CH_3_) dinuclear arene ruthenium [15] or dinuclear pentamethylcyclopentadienyl rhodium and iridium [16] complexes (see Figure 2). These dinuclear complexes bridged by embelin-derived moiety can thus be coordinated, after removal of the chlorine atoms, to N-based linear connectors (N∩N), such as pyrazine, 4,4'-bipyridine and 1,2-bis(4-pyridyl)ethylene, to generate tetranuclear tetracationic metalla-rectangles.

Herein, we present six new pentamethylcyclopentadienyl Rh(III) and Ir(III) metalla-rectangles obtained from embelin and N∩N bidentate connectors. All complexes have been characterized by infrared, ^1^H-NMR, ^13^C{^1^H}-NMR, ESI-MS and elemental analysis. The antiproliferative activity of the tetranuclear metalla-rectangles has been evaluated on cancerous (DU-145, A-549, HeLa) and noncancerous (HEK-293) cell lines. The ability of the tetracationic metalla-rectangles to accumulate in the mitochondria was confirmed by flow cytometry and confocal microscopy studies.

## 2. Results and Discussion

**Scheme 1:**
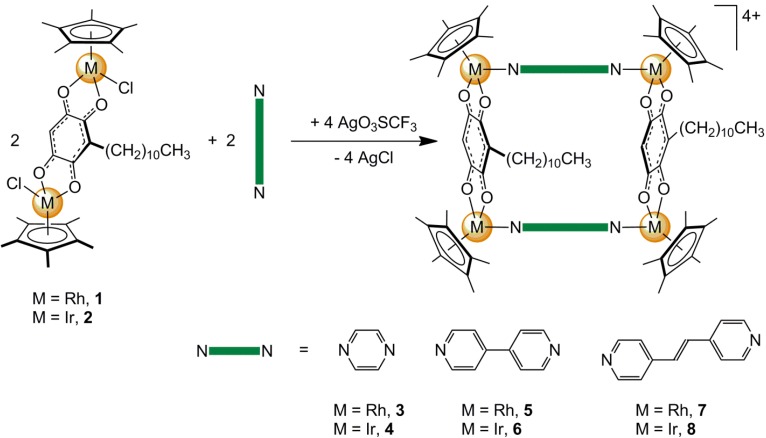
Synthesis of the metalla-rectangles [**3**](CF_3_SO_3_)_4_–[**8**](CF_3_SO_3_)_4_.

The molecular clips (η^5^-C_5_Me_5_)_2_M_2_(μ_4_-C_6_HRO_4_-κ*O*)Cl_2_ (M = Rh, **1**; M = Ir, **2**; R = (CH_2_)_10_CH_3_) were prepared by reacting in methanol the dinuclear pentamethylcyclopentadienyl complexes [(η^5^-C_5_Me_5_)M(μ-Cl)Cl]_2_ (M = Rh and Ir) with embelin (3-undecyl-2,5-dihydroxy-1,4-benzoquinone), following our previously reported method [16]. Then an equimolar reaction in methanol between the molecular clips **1**–**2** and linear ditopic ligands (N∩N) in the presence of AgCF_3_SO_3_ afforded the cationic tetranuclear metalla-rectangles of the general formula [(η^5^-C_5_Me_5_)_4_M_4_(μ_2_-N∩N-κ*N*)_2_(μ_4_-C_6_HRO_4_-κ*O*)_2_]^4+^ (N∩N = pyrazine, M = Rh, **3**; M = Ir, **4**; N∩N = 4,4'-bipyridine, M = Rh, **5**; M = Ir, **6**; N∩N = 1,2-bis(4-pyridyl)ethylene, M = Rh, **7**; M = Ir, **8**), see Scheme 1. The metalla-rectangles were isolated in high yields as their triflate salts. They were stable in air, non-hygroscopic, and they have been fully characterized by different analytical techniques, including elemental analysis. The compounds were soluble in polar solvents, insoluble in non-polar solvents, and partially soluble in water. The solubility in water was essential for further biological studies.

The ^1^H-NMR spectra of metalla-rectangles [**3**](CF_3_SO_3_)_4_–[**8**](CF_3_SO_3_)_4_ were recorded in CD_2_Cl_2_ at 25 °C and the chemical shifts of all protons have been listed in the Experimental Section. As compared to those of embelin, to the uncoordinated N∩N ligands and of the dichloro molecular clips **1** and **2**, the proton signals of the metalla-assemblies **3**–**8** were significantly shifted.

**Figure 3:**
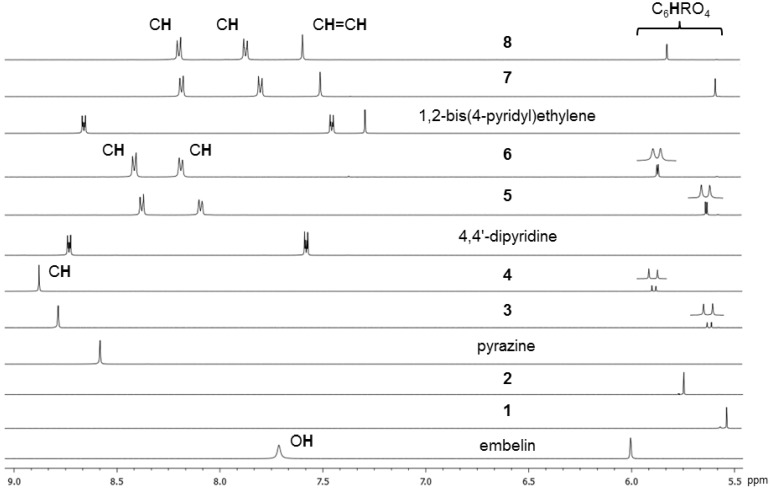
^1^H-NMR spectra of embelin, pyrazine, 4,4'-bipyridine, 1,2-bis(4-pyridyl)ethylene, metalla-clips **1**–**2** and metalla-rectangles **3**–**8** in CD_2_Cl_2_, showing only the aromatic region.

For example, the aromatic protons of the iridium derivatives (**2**, **4**, **6** and **8**) were systematically more downfield shifted than those of the rhodium analogues (**1**, **3**, **5** and **7**) (Figure 3). On the other hand, the signal associated to the methyl protons of the pentamethylcyclopentadienyl moieties was found at lower frequencies in the iridium derivatives than in the rhodium analogues.

**Figure 4:**
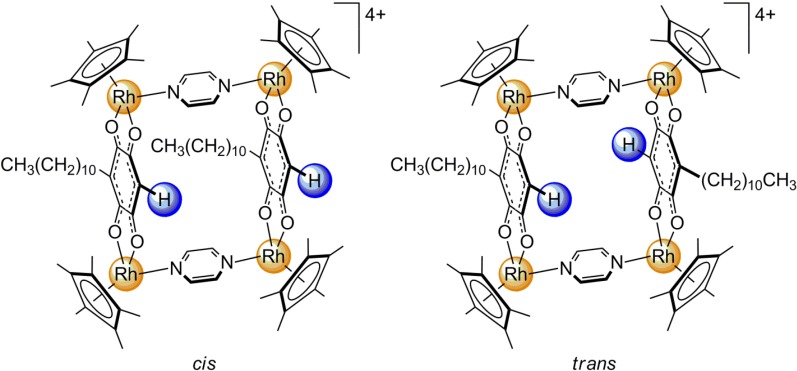
Molecular structure of the *cis*- and *trans*-isomers of the metalla-rectangle **3**.

Upon formation of the metalla-clips **1** and **2**, and of the metalla-rectangles **3**–**8**, the signal assigned to the aromatic proton of the μ_4_-C_6_**H**RO_4_-κ*O*-bridging units was always shifted upfield (5.5–5.8 ppm) as compared to the corresponding proton of embelin (~ 6 ppm). Interestingly, this aromatic proton was divided into two signals for metalla-rectangles **3**–**6**, while in the larger metalla-rectangles **7** and **8**, a singlet was observed (Figure 3). The splitting of this signal was associated to the presence of two isomers (Figure 4): a *cis*-isomer in which the undecyl chains of the μ_4_-C_6_HRO_4_-κ*O*-bridging units were parallel to each other and a *trans-*isomer in which the undecyl chains were pointing away from each other. The difference between the chemical shifts of the C_6_**H**RO_4_ proton within these two isomers was more pronounced in the pyrazine derivatives **3 ** and **4** (Δδ = 8 Hz), than in the 4,4'-bipyridine metalla-rectangles **5** and **6** (Δδ = 3 Hz). This behavior was rationalized from the relative size of these metalla-rectangles [17]. In **3** and **4** the two μ_4_-C_6_HRO_4_-κ*O* units were only separated by pyrazine linkers (M∙∙∙M ≈ 7.0 Å), thus showing a greater discrepancy between the two isomers. However, in **5** and **6** the 4,4'-bipyridine linkers increased the separation (M∙∙∙M ≈ 11.2 Å) and accordingly reduced the isomeric effect on the chemical shifts, while in **7** and **8** the 1,2-bis(4-pyridyl)ethylene linkers were long enough (M∙∙∙M ≈ 13.6 Å) that the relative orientation of the two μ_4_-C_6_HRO_4_-κ*O* units were no longer influencing the chemical shift of the C_6_**H**RO_4_ protons. The formation of these metalla-rectangles was further confirmed by electrospray ionization mass spectrometry (ESI-MS).

Electrospray ionization (ESI) mass spectra of complexes [**3**](CF_3_SO_3_)_4_–[**8**](CF_3_SO_3_)_4_ were recorded in CH_3_CN to provide further confirmation for the formation of the metalla-rectangles. All spectra showed a dicationic peak corresponding to the salt (M = [(η^5^-C_5_Me_5_)_4_M_4_(μ_2_-N∩N-κ*N*)_2_(μ_4_-C_6_HRO_4_-κ*O*)_2_](CF_3_SO_3_)_4_) after the loss of two triflate anions; [M − 2 CF_3_SO_3_]^2+^. These dicationic peaks are commonly found in the ESI mass spectra of arene ruthenium metalla-rectangles [18,19]. Furthermore, the isotopic distributions of the [M − 2 CF_3_SO_3_]^2+^ peaks were in good agreement with the expected theoretical values.

### 2.1. Biological Studies

#### MTT Assay

To understand the effect of these complexes on the proliferation of cancerous and noncancerous cells, MTT assays were carried out following the protocol reported by Wilson *et al*. [20]. The noncancerous (HEK-293) and the human cancerous cell lines (DU-145, A-549 and HeLa) were grown according to the supplier’s recommendations. All cell lines were treated for 48 h at different concentrations of complexes (0.25 µM, 0.5 µM, 0.75 µM, 1 µM, 5 µM, 10 µM, 25 µM, 50 µM and 100 µM). After treatment, the number of viable cells was estimated using a MTT assay. It was evident from these experiments that among the metalla-rectangles, [**3**](CF_3_SO_3_)_4_ exhibited the lowest IC_50_ values (around 500 nM) in the lung cancer cell line (A-549 cells), closely followed by metalla-rectangle [**8**](CF_3_SO_3_)_4_ (around 670 nM). On the other hand, the other complexes exhibited a higher number of viable cells in the studied concentration range. Cells treated with doxorubicin in the same concentration range were considered as controls. The IC_50_ values are presented in Table 1. The antiproliferative effect of the complexes on cells was clearly higher in cancer cells as compared to noncancerous cells (HEK-293), showing an excellent selectivity which reached two orders of magnitude for metalla-rectangles **3** and **8**. Since the cytotoxicity of the complexes was higher on the A-549 cells, all further cell biology experiments, like flow cytometry and confocal microscopy experiments, were performed exclusively on this cell line and with compounds [**3**](CF_3_SO_3_)_4_ and [**8**](CF_3_SO_3_)_4_.

**Table 1:** IC_50_ values (μM) of compounds [**3**](CF_3_SO_3_)_4_–[**8**](CF_3_SO_3_)_4_ on various human cancer cell lines (DU-145, A-549, HeLa) and on the noncancerous cell line HEK-293, as well as DNA melting temperatures (ΔT_m_).

Compound	Half maximum inhibitory concentration in µM (IC_50_) ^a^				ΔT_m_ (°C) ^b^
	DU-145 ^c^	A-549 ^d^	HeLa ^e^	HEK-293 ^f^	
doxorubicin	1.28 ± 0.2	0.85 ± 0.1	0.91 ± 0.2	NT	9.5
[**3**](CF_3_SO_3_)_4_	0.54 ± 0.2	0.50 ± 0.1	0.52 ± 0.2	62.0 ± 0.5	12.9
[**4**](CF_3_SO_3_)_4_	16.0 ± 0.4	3.18 ± 0.2	4.16 ± 0.3	31.2 ± 0.5	6.8
[**5**](CF_3_SO_3_)_4_	4.67 ± 0.2	0.67 ± 0.1	1.01 ± 0.3	41.3 ± 0.4	7.3
[**6**](CF_3_SO_3_)_4_	2.53 ± 0.2	0.84 ± 0.1	1.04 ± 0.3	40.3 ± 0.4	7.9
[**7**](CF_3_SO_3_)_4_	4.98 ± 0.3	2.01 ± 0.2	5.75 ± 0.2	62.1 ± 0.3	8.2
[**8**](CF_3_SO_3_)_4_	0.72 ± 0.2	0.67 ± 0.4	0.59 ± 0.2	70.8 ± 0.4	12.7

**^a^** 50% Inhibitory concentration, values averaged over three individual experiments; **^b^** Melting temperature measurements were performed after incubation for 12 h at 37 °C in PE buffer (10 mM NaH_2_PO_4_/Na_2_HPO_4_, 1 mM Na_2_EDTA) at pH 7.4 with a complex/DNA ratio of 1:1. CT DNA alone melts at 59.3 °C; **^c^** Prostate cancer; **^d^** Lung cancer; **^e^** Cervical cancer; **^f^** Normal human embryonic kidney cells; NT: Not tested.

The ability of the metalla-rectangles **3**–**8** to stabilize CT DNA (CT = calf-thymus) was confirmed by melting temperature experiments of CT DNA in the presence of the compounds. On addition of metalla-rectangles to CT DNA, a complex-DNA adduct of higher stability was evidenced by the enhancement of the melting temperature (T_m_). The melting temperature of CT DNA increased with all complexes, and especially in the presence of metalla-rectangle **3**, where the temperature shift from 59.3 °C to 72.2 °C (ΔT_m_ = 12.9 °C), indicating its potential to stabilize DNA. The melting temperatures of CT DNA (ΔT_m_) values were equally high (ΔT_m_ = 12.7 °C) with metalla-rectangle **8** (Table 1).

### 2.2. Flow Cytometry Experiments

#### 2.2.1. Cell Cycle Assay

In order to corroborate the results obtained in the MTT assays and to better understand the action of metalla-rectangles **3** and **8** on cells, the effects on the cell cycle inhibition and the interference in cell proliferations using a standard cell cycle assay were studied. The results obtained are presented in (Table 2). On analyzing the results, it was evident that the cell cycle was inhibited at the subG1 phase with both metalla-rectangles. The inhibition was more pronounced with metalla-rectangle **3** (31.97%) as compared to **8** (11.98%) or the control experiment (1.53%). Furthermore, a higher accumulation of cells in the subG1 phase upon exposure to metalla-rectangle **3**, suggested that **3 ** was capable of fragmenting DNA more efficiently than metalla-rectangle **8**.

**Table 2:** Distribution of A-549 cells in various phases of the cell cycle in the presence of [**3**](CF_3_SO_3_)_4_ and [**8**](CF_3_SO_3_)_4_ or absence of compounds (control).

Compound	Sub G1 (%)	G0/G1 (%)	S (%)	G2/M (%)
Control	1.37	54.62	17.15	16.37
[**3**](CF_3_SO_3_)_4_	31.97	56.29	2.87	1.35
[**8**](CF_3_SO_3_)_4_	11.98	66.54	7.95	3.49

#### 2.2.2. Apoptosis Assays

**Figure 5:**
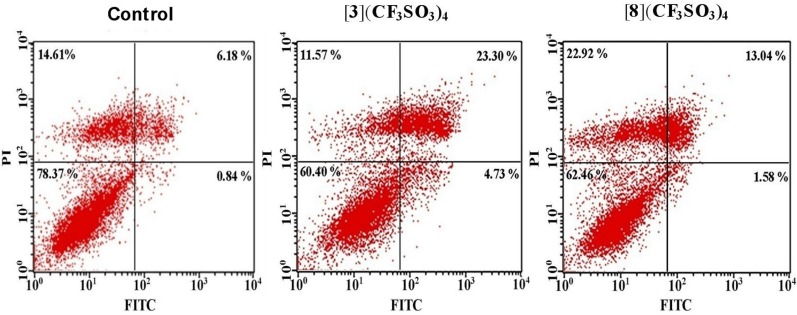
Untreated (control) and treated A-549 cells with metalla-rectangles **3** and **8** (500 nM). The cells were stained with annexin-V and propidium iodide and analyzed: viable (lower left), early apoptotic (lower right), late apoptotic (upper right), and necrotic cells (upper left).

From the MTT and cell cycle assays, it was clear that metalla-rectangle **3** was more effective in bringing down the proliferation of A-549 cells and stopping the cell cycle at the subG1 stage. To further understand the role of the metalla-rectangles **3** and **8** in inducing apoptosis in A-549 cells, an apoptosis assay was performed by treating the lung cancer cell lines with both metalla-rectangles for 48 h. The percentage of viable, necrotic, early and late apoptotic cells were estimated (Table 3 and Figure 5). 

**Table 3:** Percentages of alive, early apoptotic, late apoptotic and necrotic A-549 cells after treatments with [**3**](CF_3_SO_3_)_4_ and [**8**](CF_3_SO_3_)_4_ or without compounds (control).

Compound	Living cells (%)	Early apoptotic (%)	Late apoptotic (%)	Necrotic (%)	Total apoptotic cells (%)
Control	78.37	0.84	6.18	14.61	7.02
[**3**](CF_3_SO_3_)_4_	60.40	4.73	23.30	11.57	28.03
[**8**](CF_3_SO_3_)_4_	62.46	1.58	13.04	22.92	14.62

The results indicated that metalla-rectangle **3** effectively induced apoptosis in A-549 cells and that **3** was able to induce apoptosis in about 28.03% of cells, whereas the control and metalla-rectangle **8** induced apoptosis in respectively 7.02% and 14.62% of cells. The percentage of necrotic cells was more important with metalla-rectangle **8** (22.92%) as compared to metalla-rectangle **3** (11.57%) and the control experiment (14.61%). This suggested that metalla-rectangle **3** had a higher potential to induce apoptosis efficiently in A-549 cells when compared to metalla-rectangle **8**. Overall, the rhodium derivative [**3**](CF_3_SO_3_)_4_ was more efficient than the iridium compound [**8**](CF_3_SO_3_)_4_ in inducing apoptosis and in bringing down cancer cell proliferation in A-549 cells.

#### 2.2.3. Mitochondrial Outer Membrane Permeability

The above mentioned cell biology experiments indicated that metalla-rectangle **3** had the potential to induce apoptosis in A-549 cells, therefore its ability to alter mitochondrial outer membrane permeability was studied by mitochondria labeling with MitoTracker Red™. 

**Figure 6:**
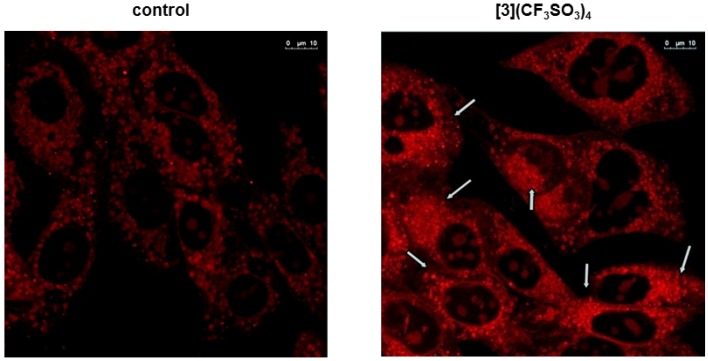
Mitochondrial outer membrane permeability in A-549 cells with (500 nM) and without (control) metalla-rectangle **3** (white arrows show aggregations of mitochondria).

It was evidenced that mitochondria were evenly distributed in the cytosol of untreated cells (see Figure 6), where about 10–15 mitochondria were seen in the cytosol. Moreover, the untreated A-549 cells (control) showed freely distributed mitochondria in the cytosol. However, upon treatment with 500 nM of metalla-rectangle **3**, aggregations of mitochondria were observed. The aggregation of mitochondria was reported to occur when cells enter into apoptosis [21], and accordingly led to the release of cytochrome c into the cytosol, which in turn triggered the cascade pathway to apoptosis. From Figure 6, it was clear that mitochondria were getting bundled with metalla-rectangle **3**, indicating the onset of apoptosis in A-549 cells.

### 2.3. DNA Binding Studies

#### 2.3.1. CD Studies

**Figure 7:**
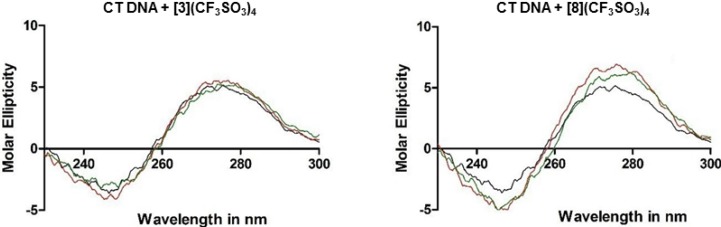
CD spectra obtained on addition of a 1:0.5 (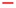) and a 1:1 (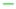) ratio of CT DNA to metalla-rectangles **3** and **8**, CT DNA alone (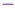).

Circular dichroism (CD) studies can provide information on the changes in the DNA conformation upon interactions with small molecules [22]. These changes in the DNA topology are giving useful information on the nature of the DNA-complex interactions [23]. On its own, the CD spectrum of CT DNA exhibits a positive band at 275 nm and a negative band at 245 nm corresponding to π–π base stacking and right-hand helicity, respectively. These bands are characteristic to the profile of the B form of DNA [24]. In the present study, the positive CD exhibited a slight hyperchromicity upon addition of metalla-rectangle **3** from 1:0.5 to 1:1, suggesting stabilization of the DNA structure. Simultaneously, the intensity of the negative band was gradually reduced, indicating a decrease in the DNA helicity. Similar interactions were observed for metalla-rectangles **5 ** and **7** after addition to CT DNA at 1:0.5 and 1:1 stoichiometries. This behavior suggests a stabilization of CT DNA [25]. When metalla-rectangle **8** was added to CT DNA, the positive CD band showed a higher hyperchromicity with an increase of the metalla-rectangle concentrations, indicating an excellent stabilization of CT DNA by metalla-rectangle **8**, see Figure 7.

#### 2.3.2. UV-Visible Spectral Studies

**Figure 8:**
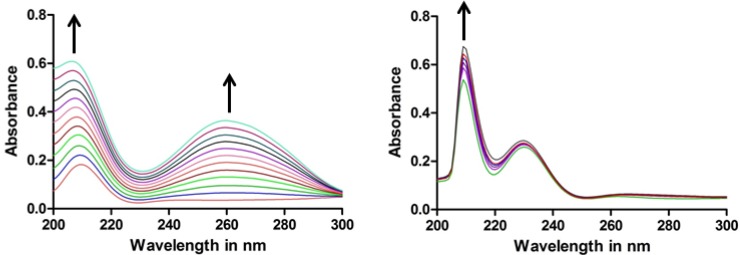
UV-visible spectra obtained with metalla-rectangles [**3**](CF_3_SO_3_)_4_ and [**8**](CF_3_SO_3_)_4_ upon interaction with CT DNA.

The mode of the DNA-complex interaction was further studied by UV-visible experiments. The results from these studies will indicate the mode of DNA-complex interaction. The UV-visible spectra of metalla-rectangles **4**, **6** and **8**, the iridium derivatives, show a prominent absorption band at 210 nm. On addition of equal increments of CT DNA to solutions of metalla-rectangles **4**, **6** and **8**, the soret band showed a continuous increase of its intensity. The hyperchromicity of the Soret band of the metalla-rectangles was more evident at 210 nm. A continuous hyperchromicity of the Soret band upon addition of metalla-rectangles **4**, **6** and **8** to a CT DNA solution is an indication of electrostatic binding [26] or partial unwinding of the CT DNA [27]. On the other hand, metalla-rectangles **3**, **5** and **7** (rhodium analogs) exhibited only an absorption band at 210 nm. However, the same hyperchromicity of the Soret band was observed upon addition of CT DNA to the metalla-rectangles, suggesting a similar mode of interactions. The UV-visible spectra of the metalla-rectangles **3** and **8** upon addition of CT DNA are shown in Figure 8.

## 3. Experimental Section

### 3.1. General

3-Undecyl-2,5-dihydroxy-1,4-benzoquinone (embelin) was purchased from ABCR GmbH (Karlsruhe, Germany) and the starting materials (η^5^-C_5_Me_5_)_2_M_2_(μ_4_-C_6_HRO_4_-ĸ*O*)Cl_2_ (M = Rh, **1**; M = Ir, **2**) were prepared according to published methods [16]. All other reagents were commercially available and used without further purification. The ^1^H and ^13^C{^1^H}-NMR spectra were recorded on a Bruker Avance II 400 MHz spectrometers using the residual protonated solvent as internal standard. Electrospray mass spectra were obtained in positive-ion mode with a Bruker FTMS 4.7T BioAPEX II mass spectrometer, University of Fribourg (Switzerland). Infrared spectra were recorded as KBr pellets with a Perkin-Elmer FTIR 1720 X spectrometer. Microanalyses were carried out by the Mikroelementaranalytisches Laboratorium, ETH Zürich (Switzerland). 

*[(η^5^-C_5_Me_5_)_4_Rh_4_(μ_2_-pyrazine-ĸN)_2_(μ_4_-C_6_HRO_4_-ĸO)_2_](CF_3_SO_3_)_4_* ([**3**](CF_3_SO_3_)_4_). A mixture of **1** (100 mg, 0.14 mmol) and AgCF_3_SO_3_ (72 mg, 0.28 mmol) in methanol (25 mL) was stirred at room temperature for 3 h and then filtered to remove AgCl. Pyrazine (11.12 mg, 0.14 mmol) was added to the filtrate and stirred for 15 h at room temperature. The solvent was removed under reduced pressure and dichloromethane (3 mL) was added. Addition of diethyl ether (100 mL) to the dichloromethane solution gave the desired product as a green powder. Yield 120 mg (75%). Calcd for C_86_H_116_N_4_O_20_F_12_S_4_Rh_4_·2H_2_O: C, 44.34; H, 5.19; N, 2.40. Found: C, 44.23; H, 5.12; N, 2.20. ESI MS (CH_3_CN): *m/z* = 958.19 [(η^5^-C_5_Me_5_)_2_Rh_2_(μ_4_-C_6_HRO_4_-κ*O*) + CH_3_CN + CF_3_SO_3_]^+^. IR (KBr pellets): *ν* = 3468 (s, C−H_ar_), 2926 (s, C−H), 2855 (s, C−H), 1522 (s, C=O), 1261 cm^−1^ (s, CF_3_). ^1^H-NMR (400 MHz, CD_2_Cl_2_): δ = 8.76 (s, 8H, C**H**_pyrazine_), 5.62 (s, 1H, C**H**_embelin_), 5.60 (s, 1H, C**H**_embelin_), 2.38 (m, 4H, C**H**_2_(CH_2_)_9_CH_3_), 1.73 (s, 60H, C_5_(C**H**_3_)_5_), 1.35 (m, 36H, CH_2_(C**H**_2_)_9_CH_3_), 0.93 (t, 6H, *^3^J* = 8 Hz, CH_2_(CH_2_)_9_C**H**_3_) ppm. ^13^C{^1^H}-NMR (100 MHz, CD_2_Cl_2_): δ = 184.2, 181.9, 149.9, 116.8, 97.2, 32.4, 30.2, 30.1, 29.8, 23.1, 14.3, 8.8 ppm.

*[(η^5^-C_5_Me_5_)_4_Ir_4_(μ_2_-pyrazine-ĸN)_2_(μ_4_-C_6_HRO_4_-ĸO)_2_](CF_3_SO_3_)_4_* ([**4**](CF_3_SO_3_)_4_). A mixture of **2** (100 mg, 0.11 mmol) and AgCF_3_SO_3_ (58 mg, 0.22 mmol) in methanol (25 mL) was stirred at room temperature for 3 h and then filtered to remove AgCl. Pyrazine (9 mg, 0.11 mmol) was added to the filtrate and stirred for 15 h at room temperature. The solvent was removed under reduced pressure and dichloromethane (3 mL) was added. Addition of diethyl ether (100 mL) to the dichloromethane solution gave the desired product as a brown powder. Yield 130 mg (89%). Calcd for C_86_H_116_N_4_O_20_F_12_S_4_Ir_4_ 2 (C_2_H_5_)_2_O: C, 40.33; H, 4.90; N, 2.00. Found: C, 40.54; H, 4.50; N, 1.90. ESI MS (CH_3_CN): *m/z* = 1095.29 [M − 2 CF_3_SO_3_]^2+^. IR (KBr pellets): *ν* = 3468 (s, C−H_ar_), 2926 (s, C−H), 2855 (s, C−H), 1525 (s, C=O), 1259 cm^−1^ (s, CF_3_). ^1^H-NMR (400 MHz, CD_2_Cl_2_): δ = 8.87 (s, 8H, C**H**_pyrazine_), 5.89 (s, 1H, C**H**_embelin_), 5.87 (s, 1H, C**H**_embelin_), 2.47 (m, 4H, C**H**_2_(CH_2_)_9_CH_3_), 1.68 (s, 60H, C_5_(C**H**_3_)_5_), 1.32 (m, 36H, CH_2_(C**H**_2_)_9_CH_3_), 0.92 (t, 6H, *^3^J* = 8 Hz, CH_2_(CH_2_)_9_C**H**_3_) ppm. ^13^C{^1^H}-NMR (100 MHz, CD_2_Cl_2_): δ = 185.8, 183.6, 151.4, 110.4, 89.0, 30.3, 30.2, 30.1, 29.8, 23.1, 14.3, 8.8 ppm.

*[(η^5^-C_5_Me_5_)_4_Rh_4_(μ_2_-4,4'-bipyridine-ĸN)_2_(μ_4_-C_6_HRO_4_-ĸO)_2_](CF_3_SO_3_)_4_* ([**5**](CF_3_SO_3_)_4_). A mixture of **1** (100 mg, 0.14 mmol) and AgCF_3_SO_3_ (72 mg, 0.28 mmol) in methanol (25 mL) was stirred at room temperature for 3 h and then filtered to remove AgCl. 4,4'-bipyridine (22 mg, 0.14 mmol) was added to the filtrate and stirred for 15 h at room temperature. The solvent was removed under reduced pressure and dichloromethane (3 mL) was added. Addition of diethyl ether (100 mL) to the dichloromethane solution gave the desired product as a green powder. Yield 140 mg (82%). Calcd for C_98_H_124_N_4_O_20_F_12_S_4_Rh_4_·2H_2_O: C, 47.42; H, 5.20; N, 2.26. Found: C, 47.34; H, 5.16; N, 2.08. ESI MS (CH_3_CN): *m/z* = 1073.23 [M − 2 CF_3_SO_3_]^2+^. IR (KBr pellets): *ν* = 3498 (s, C−H_ar_), 2925 (s, C−H), 2854 (s, C−H), 1517 (s, C=O), 1261 cm^−1^ (s, CF_3_). ^1^H-NMR (400 MHz, CD_2_Cl_2_): δ = 8.36 (d, 8H, *^3^J* = 8 Hz, C**H**_bipy_), 8.08 (d, 8H, *^3^J* = 8 Hz, C**H**_bipy_), 5.63 (s, 1H, C**H**_embelin_), 5.62 (s, 1H, C**H**_embelin_), 2.39 (m, 4H, C**H**_2_(CH_2_)_9_CH_3_), 1.68 (s, 60H, C_5_(C**H**_3_)_5_), 1.36 (m, 36H, CH_2_(C**H**_2_)_9_CH_3_), 0.93 (t, 6H, *^3^J* = 8 Hz, CH_2_(CH_2_)_9_C**H**_3_) ppm. ^13^C{^1^H}-NMR (100 MHz, CD_2_Cl_2_): δ = 184.2, 181.8, 152.4, 145.5, 125.0, 96.0, 96.0, 32.4, 30.2, 30.0, 29.8, 29.0, 23.1, 8.9 ppm.

*[(η^5^-C_5_Me_5_)_4_Ir_4_(μ_2_-4,4'-bipyridine-ĸN)_2_(μ_4_-C_6_HRO_4_-ĸO)_2_](CF_3_SO_3_)_4_* ([**6**](CF_3_SO_3_)_4_). A mixture of **2** (100 mg, 0.11 mmol) and AgCF_3_SO_3_ (58 mg, 0.22 mmol) in methanol (25 mL) was stirred at room temperature for 3 h and then filtered to remove AgCl. 4,4'-bipyridine (18 mg, 0.11 mmol) was added to the filtrate and stirred for 15 h at room temperature. The solvent was removed under reduced pressure and dichloromethane (3 mL) was added. Addition of diethyl ether (100 mL) to the dichloromethane solution gave the desired product as a brown powder. Yield 130 mg (84%). Calcd for C_98_H_124_N_4_O_20_F_12_S_4_Ir_4_·2CH_2_Cl_2_·H_2_O: C, 40.16; H, 4.38; N, 1.87. Found: C, 40.01; H, 4.24; N, 1.83. ESI MS (CH_3_CN): *m/z* = 1251.34 [M − 2 CF_3_SO_3_]^2+^. IR (KBr pellets): *ν* = 3469 (s, C−H_ar_), 2925 (s, C−H), 2853 (s, C−H), 1524 (s, C=O), 1260 cm^−1^ (s, CF_3_). ^1^H-NMR (400 MHz, CD_2_Cl_2_): δ = 8.39 (d, 8H, *^3^J* = 4 Hz, C**H**_bipy_), 8.16 (d, 8H, *^3^J* = 4 Hz, C**H**_bipy_), 5.86 (s, 1H, C**H**_embelin_), 5.85 (s, 1H, C**H**_embelin_), 2.46 (m, 4H, C**H**_2_(CH_2_)_9_CH_3_), 1.64 (s, 60H, C_5_(C**H**_3_)_5_), 1.34 (m, 36H, CH_2_(C**H**_2_)_9_CH_3_), 0.93 (t, 6H, *^3^J* = 8 Hz, CH_2_(CH_2_)_9_C**H**_3_) ppm. ^13^C{^1^H}-NMR (100 MHz, CD_2_Cl_2_): δ = 185.7, 183.5, 152.8, 145.3, 125.5, 87.7, 32.3, 30.1, 30.1, 29.9, 29.8, 28.7, 23.1, 14.3, 9.0 ppm.

*[(η^5^-C_5_Me_5_)_4_Rh_4_(μ_2_-1,2-bis(4-pyridyl)ethylene-ĸN)_2_(μ_4_-C_6_HRO_4_-ĸO)_2_](CF_3_SO_3_)_4_* ([**7**](CF_3_SO_3_)_4_). A mixture of **1** (100 mg, 0.14 mmol) and AgCF_3_SO_3_ (72 mg, 0.28 mmol) in methanol (25 mL) was stirred at room temperature for 3 h and then filtered to remove AgCl. 1,2-Bis(4-pyridyl)ethylene (26 mg, 0.14 mmol) was added to the filtrate and stirred for 15 h at room temperature. The solvent was removed under reduced pressure and dichloromethane (3 mL) was added. Addition of diethyl ether (100 mL) to the dichloromethane solution gave the desired product as a green powder. Yield 120 mg (69%). Calcd for C_102_H_128_N_4_O_20_F_12_S_4_Rh_4_·2CH_2_Cl_2_: C, 46.82; H, 4.99; N, 2.10. Found: C, 46.81; H, 5.01; N, 1.90. ESI MS (CH_3_CN): *m/z* = 1099.24 [M − 2 CF_3_SO_3_]^2+^. IR (KBr pellets): *ν* = 3480 (s, C−H_ar_), 2925 (s, C−H), 2854 (s, C−H), 1520 (s, C=O), 1261 cm^−1^ (s, CF_3_). ^1^H-NMR (400 MHz, CD_2_Cl_2_): δ = 8.17 (d, 8H, *^3^J* = 4 Hz, C**H**_pyridyl_), 7.79 (d, 8H, *^3^J* = 8 Hz, C**H**_pyridyl_), 7.50 (s, 4H, C**H**=C**H**), 5.59 (s, 2H, C**H**_embelin_), 2.36 (m, 4H, C**H**_2_(CH_2_)_9_CH_3_), 1.67 (s, 60H, C_5_(C**H**_3_)_5_), 1.35 (m, 36H, CH_2_(C**H**_2_)_9_CH_3_), 0.90 (t, 6H, *^3^J* = 8 Hz, CH_2_(CH_2_)_9_C**H**_3_) ppm. ^13^C{^1^H}-NMR (100 MHz, CD_2_Cl_2_): δ = 184.1, 181.8, 151.5, 146.8, 132.0, 125.3, 116.5, 101.4, 95.8, 32.3, 30.2, 30.2, 30.1, 29.8, 23.1, 14.3, 9.0 ppm.

*[(η^5^-C_5_Me_5_)_4_Ir_4_(μ_2_-1,2-bis(4-pyridyl)ethylene-ĸN)_2_(μ_4_-C_6_HRO_4_-ĸO)_2_](CF_3_SO_3_)_4_* ([**8**](CF_3_SO_3_)_4_). A mixture of **2** (100 mg, 0.11 mmol) and AgCF_3_SO_3_ (58 mg, 0.22 mmol) in methanol (25 mL) was stirred at room temperature for 3 h and then filtered to remove AgCl. 1,2-bis(4-pyridyl)ethylene (21 mg, 0.11 mmol) was added to the filtrate and stirred for 15 h at room temperature. The solvent was removed under reduced pressure and dichloromethane (3 mL) was added. Addition of diethyl ether (100 mL) to the dichloromethane solution gave the desired product as a brown powder. Yield 130 mg (83%). Calcd for C_102_H_128_N_4_O_20_F_12_S_4_Ir_4 _∙ 6 CH_2_Cl_2_: C, 38.55; H, 4.19; N, 1.67. Found: C, 38.36; H, 4.44; N, 1.99. ESI MS (CH_3_CN): *m/z* = 1279.36 [M − 2 CF_3_SO_3_]^2+^. IR (KBr pellets): *ν* = 3468 (s, C−H_ar_), 2925 (s, C−H), 2853 (s, C−H), 1522 (s, C=O), 1262 cm^−1^ (s, CF_3_). ^1^H-NMR (400 MHz, CD_2_Cl_2_): δ = 8.18 (d, 8H, *^3^J* = 4 Hz, C**H**_pyridyl_), 7.85 (d, 8H, *^3^J* = 4 Hz, C**H**_pyridyl_), 7.58 (s, 4H, C**H**=C**H**), 5.81 (s, 2H, C**H**_embelin_), 2.48 (m, 4H, C**H**_2_(CH_2_)_9_CH_3_), 1.63 (s, 60H, C_5_(C**H**_3_)_5_), 1.34 (m, 36H, CH_2_(C**H**_2_)_9_CH_3_), 0.91 (t, 6H, *^3^J* = 8 Hz, CH_2_(CH_2_)_9_C**H**_3_) ppm. ^13^C{^1^H}-NMR (100 MHz, CD_2_Cl_2_): δ = 185.6, 183.5, 151.8, 147.1, 132.2, 125.8, 116.5, 110.4, 87.4, 32.3, 30.2, 30.1, 29.9, 29.8, 28.6, 23.1, 22.4, 14.3, 9.1 ppm.

### 3.2. MTT Assay

The anticancer activity of the complex was determined using the 3-(4,5-dimethylthiazol-2-yl)-2,5-diphenyltetrazolium bromide (MTT) assay [28]. First 1 × 10^4^ cells per well were seeded in Dulbecco’s modified Eagle’s medium (DMEM, 100 µL) supplemented with 10% fetal bovine serum in each well of 96-well microculture plates and incubated for 24 h at 37 °C in a CO_2_ incubator. Complexes were diluted to the desired concentrations in culture medium and added to the wells with respective vehicle control. After 48 h of incubation, MTT (10 µL, 5 mg mL^−1^) was added to each well and the plates were further incubated for 4 h. Then the supernatant from each well was carefully removed, formazan crystals were dissolved in DMSO (100 µL) and absorbance at 570 nm was recorded.

### 3.3. Flow Cytometry Study

Flow cytometric analysis (FACS) was performed to evaluate the distribution of the cells in different cell cycle phases. A-549 cells were incubated with metalla-rectangles **3** and **8** at 500 nM concentration for 24 h. Untreated cells were considered as control sample. Untreated and treated cells were harvested, washed with PBS, fixed in ice cold 70% ethanol and stained with propidium iodide (Sigma Aldrich, St. Louis, MO, USA). Cell cycle was performed by using Becton Dickinson FACS Caliber (Franklin Lakes, NJ, USA).

After 90% confluence, the A-549 cells were treated with 500 nM of [**3**](CF_3_SO_3_)_4_ and [**8**](CF_3_SO_3_)_4_ respectively, for 3 h. Then 1 × 10^6^ cells were washed with 2× binding buffer and re-suspended in the binding buffer (100 µL) and Annexin-V-fluorescein isothiocyanate (FITC) (1.0 µg). Untreated cells were considered as control. The cells were incubated at room temperature for 10 min, followed by addition of a binding buffer (400 µL) containing PI (1 µL). Stained cells were analyzed using a FACS Calibur flow cytometer from B. D. Biosciences (USA). Annexin-V FITC conjugated and PI-labelled cells were excited using a 488 nm solid-state laser and the fluorescence emission intensity was captured using 530/30 and 585/42 band-pass filters, respectively.

### 3.4. UV-Visible Spectroscopic Titrations

UV-Visible spectroscopic titrations were performed using an ABI Lambda 40 UV/Vis spectrophotometer (Foster City, CA, USA) at 25 °C using 1 cm path length quartz cuvettes. Stock solutions of complex (25 µM) was prepared in DMSO and CT DNA (25 µM) were prepared in KBPES buffer (30 mM Potassium Phosphate with 100 mM KCl, pH 7.0). The quartz cells were thoroughly cleaned with distilled water and followed by nitric acid (~0.1 N) after each experiment. UV-visible absorption titrations were done by adding CT DNA stock solution (25 µM) in KBPES buffer (30 mM Potassium Phosphate with 100 mM KCl, pH 7.0) to the quartz cuvette containing approximately 25 µM complex solutions. Preparation of CT DNA and complex solutions was done on the same day as the experiment. Titrations were performed until the band remained at a fixed wavelength upon successive additions of CT DNA. 

### 3.5. CD Spectroscopic Study

Circular dichroism (CD) experiments were performed using a Jasco 815 CD spectropolarimeter (Jasco, Tokyo, Japan). CD spectra were recorded from 230 to 300 nm to find the conformational changes in the CT DNA after interaction with the complexes. For each CD experiment, CT DNA (15 × 10^‒6^ M) was used initially. Further, for the characterization of complex‒DNA interaction, the CD spectra were recorded in 1:0.5 and 1:1 CT DNA/complex molar ratios. CD titrations were performed in KBPES buffer (30 mM Potassium Phosphate with 100 mM KCl, pH 7.0) at 25 °C. Each spectrum was recorded three times and the average of three scans was taken.

### 3.6. Thermal Denaturation Study

The DNA binding affinity of the synthesized complexes was evaluated through thermal denaturation studies with duplex CT DNA using a modified reported procedure [29]. Working solutions in aqueous buffer (10 mM NaH_2_PO_4_/Na_2_HPO_4_, 1 mM Na_2_EDTA, pH 7.4) containing CT DNA (100 µM) and the complexes (20 µM) were prepared by addition of concentrated complex solutions in 1:1 stoichiometry to obtain a fixed molar ratio of 1:5. The DNA-hybrid solutions are incubated at 37 °C for 12 h prior to analysis. Samples were monitored at 260 nm using a Beckman DU-7400 spectrophotometer (Pasadena, CA, USA) fitted with high-performance temperature controller and were heated at 1 °C min^−1^ in the range of 40–95 °C.

### 3.7. Mitochondria Outer Membrane Permeability Assay

Compound [**3**](CF_3_SO_3_)_4_ (500 nM) with treated and untreated A-549 cells were incubated in a medium containing MitoTracker Red™ (50 nM) for 30 min at culture conditions recommended by the supplier. Cells were washed and fixed with 4% paraformaldehyde for 15 min, and permeabilized with Triton X-100 (0.1%) for each 10 min. The cover glasses after PBS wash were mounted on to the glass slides, sealed with nail polish and observed using laser scanning confocal microscopy (Leica TCS SP5, Heidelberg, Germany). The MitoTracker Red™ was excited using 543 nm laser source and the fluorescence emission signals were collected at 570 nm to 635 nm.

## 4. Conclusions

A series of half-sandwich rhodium and iridium metalla-rectangles has been prepared and evaluated as anticancer agents. All tetranuclear complexes show an exceptional selectivity for cancerous over noncancerous cell lines. This selectivity is reaching two orders of magnitude for the most active compound, the rhodium pyrazine derivative [(η^5^-C_5_Me_5_)_4_Rh_4_(μ_2_-pyrazine-κ*N*)_2_(μ_4_-C_6_HRO_4_-κ*O*)_2_]^4+^ ([**3**]^4+^), which shows an IC_50 _of 0.5 μM on the cancerous cell lines DU-145, A-549 and HeLa but only of 62 μM on the noncancerous cells HEK-293. These metalla-rectangles appear to interact with DNA and with the outer membrane of mitochondria, most likely because of the positive charge of the metalla-rectangles and of the presence of lipophilic side chains, thus conferring to these metalla-assemblies the necessary features for an *in vivo* study.
